# Autoimmune hemolytic anemia in the era of immunotherapy: from pathogenesis to therapeutic strategies

**DOI:** 10.1186/s12967-026-08787-7

**Published:** 2026-08-10

**Authors:** Sijia Yan, Jiaying Wu, Mi Zhou, Yi Xiao

**Affiliations:** Department of Hematology, Tongji Hospital, Tongji Medical College, Huazhong University of Science and Technology, Wuhan, 430030 China

**Keywords:** Autoimmune hemolytic anemia, Warm autoimmune hemolytic anemia, Cold agglutinin disease, Complement inhibitor, Spleen tyrosine kinase

## Abstract

Autoimmune hemolytic anemia (AIHA) is a rare hematologic disorder characterized by the accelerated destruction of red blood cells mediated by autoantibodies and is classified into warm antibody type and cold antibody type. Corticosteroids remain the first-line treatment, whereas rituximab has replaced splenectomy as the standard second-line therapy. However, a considerable proportion of patients still experience relapse or refractory disease. In recent years, with an improved understanding of its pathogenesis, significant advances have been achieved in targeted therapies for AIHA, including complement inhibitors, spleen tyrosine kinase inhibitors, Bruton tyrosine kinase inhibitors, phosphoinositide 3-kinase delta inhibitors, other novel agents, and chimeric antigen receptor T-cell therapy. Future AIHA management will focus on individualized treatment guided by precise stratification, combination strategies, and biomarkers, ultimately achieving a transition from empirical immunosuppression to mechanism-targeted precision medicine.

## Introduction

Autoimmune hemolytic anesmia (AIHA) is a rare hematologic disorder characterized by accelerated red blood cell (RBC) destruction mediated by autoantibodies, with or without complement involvement. Based on the optimal reaction temperature of autoantibodies to RBCs, AIHA is classified into warm antibody type (wAIHA), cold antibody type (cAIHA), and mixed type (mAIHA), with wAIHA being the most common subtype, accounting for approximately 60–70% of all cases [1]. cAIHA is further classified into cold agglutinin disease (CAD), cold agglutinin syndrome (CAS), and paroxysmal cold hemoglobinuria [2] (Table 1).

**Table 1:** Main differences between wAIHA and cAIHA

Feature	wAIHA	cAIHA
Predominant autoantibody	IgG	IgM
Thermal reactivity	Active at 37 °C	Active at low temperatures (< 30 °C)
Dominant effector mechanism	Fcγ receptor–mediated extravascular hemolysis	Complement-mediated hemolysis
Complement involvement	Variable, often limited to C3 deposition	Central and sustained activation of classical pathway
Site of RBC destruction	Spleen, liver (macrophage-rich organs)	Liver + intravascular (terminal complement activation)
First-line therapy	Corticosteroids	Avoidance of cold exposure; steroids usually ineffective
Steroid efficacy	High	Poor or transient
Rituximab efficacy	Moderate, relapse common	Good, more durable responses
Role of complement inhibitors	Limited/selected cases	Core therapeutic strategy
Pathogenic driver	Fc receptor–driven phagocytosis	Classical complement cascade activation
Key emerging therapies	BTK, Syk, PI3Kδ inhibitors	C1s, C3, factor B inhibitors

A fundamental mechanistic distinction exists between wAIHA and cAIHA. wAIHA is predominantly mediated by IgG autoantibodies that bind red blood cells at 37 °C and promote Fcγ receptor–dependent phagocytosis by splenic and hepatic macrophages, with variable and often secondary complement activation. In contrast, cAIHA is driven primarily by IgM autoantibodies with high thermal amplitude variability that efficiently activate the classical complement cascade, resulting in C3-mediated opsonization and, in severe cases, terminal complement–driven intravascular hemolysis [3]. These differences define two distinct effector axes: an Fc receptor–dominant pathway in wAIHA and a complement-dominant pathway in cAIHA.

These mechanistic divergences translate into distinct therapeutic responses. Corticosteroids remain first-line therapy in wAIHA, achieving response rates of approximately 70–80%, although relapse during tapering is common. By contrast, cAIHA exhibits limited and typically transient responses to corticosteroids, reflecting its complement-driven pathophysiology [4]. Rituximab demonstrates clinical activity in both subtypes but tends to produce more durable disease control in cAIHA. More recently, complement-targeting therapies have shown pronounced efficacy in cAIHA, consistent with its central dependence on complement activation, whereas their benefit in wAIHA is more variable and context-dependent [5].

**Fig. 1:**
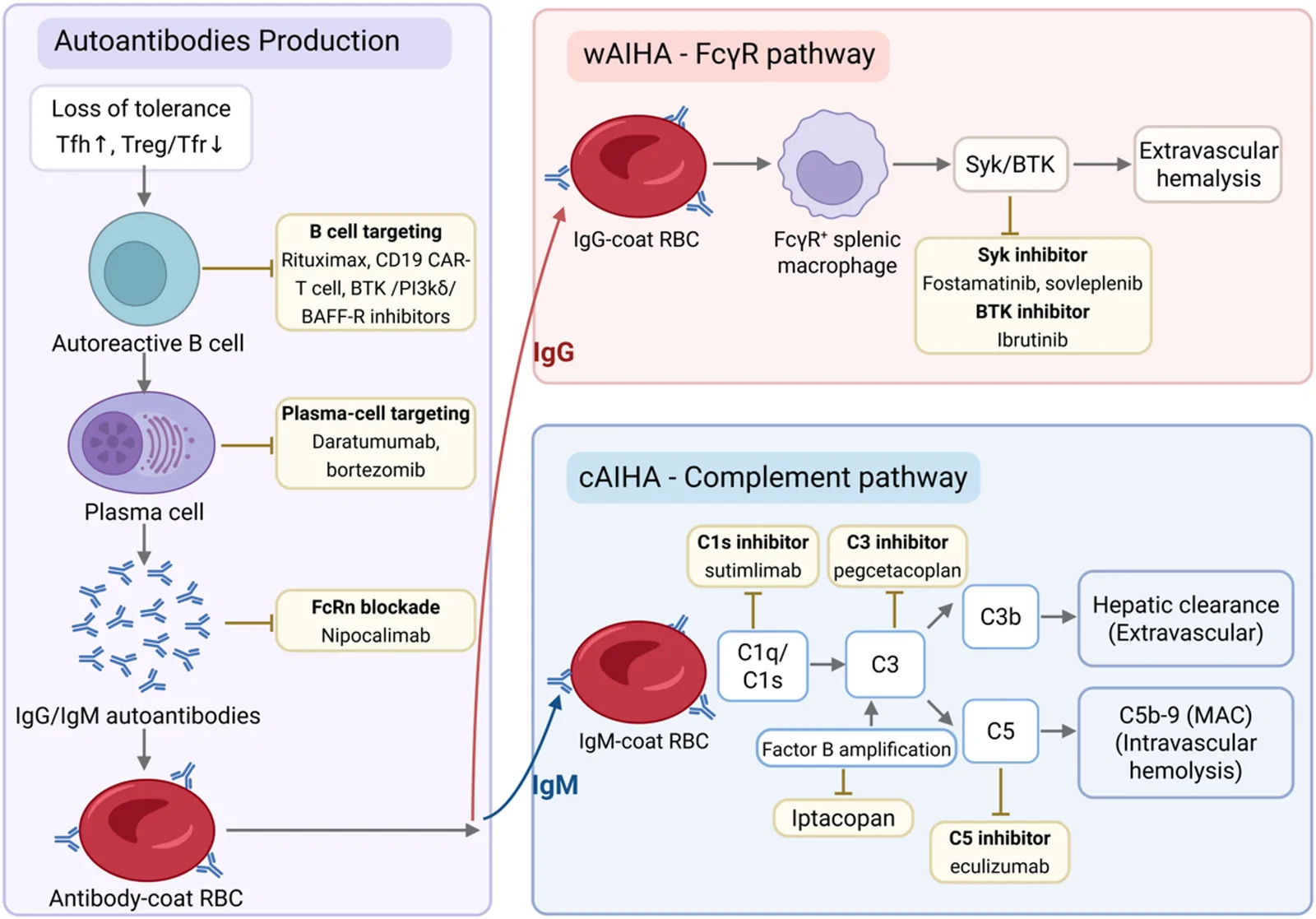
Targeted therapies for autoimmune hemolytic anemia: mechanisms of action

Together, these observations support a model in which wAIHA is primarily driven by IgG–Fc receptor–mediated extravascular hemolysis with partial complement amplification, whereas cAIHA represents a complement-amplified hemolytic disorder dominated by IgM-mediated classical pathway activation. This mechanistic dichotomy provides a rational framework for subtype-specific therapeutic strategies and underpins the development of emerging targeted therapies across B-cell, plasma cell, and complement pathways. In parallel, the therapeutic landscape of AIHA has expanded substantially with the advent of complement inhibitors, B-cell–directed agents, plasma cell–targeted therapies, and intracellular signaling modulators, reshaping treatment beyond conventional immunosuppression (Fig. 1).

### Immunopathogenesis of AIHA: cellular and molecular drivers

AIHA is the ultimate consequence of a broad disruption of immune resistance. This pathological process involves the participation of autoreactive B cells and plasma cells, dysregulated CD4⁺ T cell subsets, innate immunity effector cells, inflammatory cytokines, and the complement system. The contributions of distinct immune components differ between w AIHA and cAIHA, thereby accounting for the differences in their clinical manifestations and therapeutic sensitivities.

Self-reactive B cells are the primary source of pathogenic autoantibodies. Upon recognition of red blood cell antigens by the B cell receptor (BCR), B cell activation, survival, and differentiation are promoted through the Syk, Bruton’s tyrosine kinase (BTK), and PI3K-AKT-mTOR signaling pathways. Elevated levels of B cell activating factor (BAFF) enable self-reactive B cells that have escaped negative selection to persist by activating both the classical and non-canonical NF-κB pathways, which are dependent on the BAFF receptor [6]. In experimental AIHA, CD4⁺CXCR5⁺CD25⁻Foxp3⁻ T follicular helper (Tfh) cells are significantly expanded and exhibit high expression of inducible T-cell costimulator and programmed cell death protein 1 (PD-1). Conversely, CD4⁺CXCR5⁺CD25⁺Foxp3⁺ T follicular regulatory (Tfr) cells are relatively diminished, resulting in an elevated Tfh/Tfr ratio, which is correlated with increased levels of autoantibodies. Concurrently, elevated levels of interleukin-6 (IL-6) and IL-21, along with upregulated mRNA expression of B-cell lymphoma 6 (Bcl-6), c-Maf, and IL-21, suggest that excessive follicular helper activity promotes B cell activation, class switching, and production of anti-erythrocyte autoantibodies. Long-lived plasma cells, generated through the germinal center reaction, can sustain antibody secretion independently of circulating CD20⁺ B cells, which may explain disease relapse following B cell depletion therapy and provides a rationale for targeting plasma cells in the treatment of refractory AIHA [7].

Immune resistance mediated by T cells constitutes a critical component of the breakdown of tolerance of self in AIHA [7]. Regulatory T cells (Tregs), which typically express CD4, high levels of CD25, and the transcription factor Foxp3, maintain immune homeostasis through cytotoxic T-lymphocyte-associated protein 4 (CTLA-4)-mediated inhibitory effects and the secretion of interleukin-10 (IL-10) and transforming growth factor-beta (TGF-β) [8]. In wAIHA, inflammation induced by persistent hemolysis may further exacerbate T-cell immune imbalance. Following the phagocytosis of IgG-coated erythrocytes, monocytes and macrophages can produce substantial amounts of tumor necrosis factor-alpha (TNF-α), which may serve as a key inflammatory mediator linking sustained hemolysis with the disruption of immune resistance. Studies have demonstrated that the TNF signaling pathway is significantly activated in Tregs of patients with wAIHA, and the degree of activation is inversely correlated with the proportion and absolute count of circulating Tregs, as well as Foxp3 protein expression levels. Within the inflammatory microenvironment of wAIHA, persistently enhanced TNF signaling may promote post-translational destabilization of Foxp3 through activation of the non-canonical NF-κB pathway, involving molecules such as DBC1 and caspase-8, thereby impairing the immunosuppressive function of Tregs [9]. These findings suggest that the reduction in Treg numbers, along with impaired functional capacity and diminished stability, may collectively drive autoreactive immune responses in AIHA and contribute to the persistence of autoantibody production and hemolysis.

The aforementioned immune networks ultimately converge into distinct effector mechanisms across different subtypes of AIHA. In wAIHA, IgG autoantibodies bind to red blood cells at 37 °C, predominantly mediating extravascular hemolysis via Fcγ receptors, accompanied by varying degrees of C3 deposition [10]. In contrast, in CAD, clonal B cells in the bone marrow produce cold-reactive monoclonal IgM. These antibodies bind to red blood cells in cooler peripheral tissues, efficiently recruiting C1q and sequentially activating C1r/C1s, C4, C2, and C3. Upon the return of blood to the warmer central circulation, IgM may dissociate from the erythrocyte surface, while C3 fragments remain deposited, thereby facilitating recognition and clearance by hepatic macrophages. If the terminal complement pathway is fully activated, the C5b-9 membrane attack complex can be formed, further inducing intravascular hemolysis [11].

Collectively, AIHA results from a coordinated breakdown of B- and T-cell immune tolerance that sustains anti-erythrocyte autoantibody production and activates Fcγ receptor- and complement-dependent effector pathways, thereby driving subtype-specific patterns of hemolysis and differential therapeutic responses.

### wAIHA

wAIHA may be primary or secondary to hematologic malignancies (such as non-Hodgkin lymphoma and chronic lymphocytic leukemia), infectious diseases, immunodeficiency, or drug therapy (such as immune checkpoint inhibitors) [12]. It is widely accepted that polyclonal IgG binds to RBC antigens, such as Rh or Band 3, at 37 °C. IgG-coated RBCs subsequently bind to Fc gamma (Fcγ) receptors on splenic macrophages, leading to extravascular hemolysis through antibody-dependent cellular phagocytosis and antibody-dependent cellular cytotoxicity. Concurrently, activation of the classical complement pathway may result in mild intravascular hemolysis. Its initiation is associated with T helper 17 (Th17)/regulatory T cell imbalance, infection, or drug-induced disruption of immune tolerance, ultimately contributing to the development of wAIHA [5]. The direct antiglobulin test (DAT) is the cornerstone for diagnosing wAIHA. For DAT-negative patients, high-sensitivity methods such as flow cytometry or assessment of response to corticosteroid therapy should be used to confirm the diagnosis [13].

### First-line treatment

Glucocorticoids remain the first-line therapy for wAIHA. More than 80% of newly diagnosed patients respond to prednisone treatment. In responders, the dose should be gradually tapered and discontinued after 4–6 weeks of therapy. However, a considerable proportion of patients relapse during dose tapering or after treatment discontinuation [14]. Studies have shown that combination therapy with glucocorticoids and rituximab is more effective than glucocorticoid monotherapy. In the RAIHA study, the 1-year overall response rate and the 2-year sustained complete response rate in patients treated with rituximab combined with short-term prednisone were significantly higher than those in patients receiving prednisone alone, with lower mortality and severe infection rates. This study provided the first evidence supporting the safety and efficacy of rituximab combined with corticosteroids in newly diagnosed wAIHA [15]. For patients with severe anemia or those who are not suitable candidates for high-dose corticosteroids, first-line treatment may include the combination of corticosteroids and rituximab.

### Second-line treatment

For patients who are refractory to, relapse after, intolerant of, or dependent on glucocorticoids, second-line therapy should be considered. Before the introduction of rituximab, splenectomy was the standard second-line treatment for wAIHA. However, splenectomy significantly increases the risk of severe infection and thromboembolism [16]. Reynaud et al. analyzed 409 patients with AIHA and reported that the overall response rate to rituximab monotherapy in wAIHA was 79%, with a complete remission rate of 40%. The peak response was achieved 2–4 months after treatment initiation, with a complete remission rate of 70%. Regarding safety, 14% of patients experienced adverse events, most commonly infusion-related fever and chills. The incidence of serious adverse events was approximately 6%, and the overall mortality rate during follow-up was 4.6% [17]. The favorable safety and efficacy profile of rituximab has established it as the preferred second-line therapy for wAIHA, thereby reshaping its treatment paradigm.

Calcineurin inhibitors, including cyclosporine and tacrolimus, are also used in patients who are not suitable candidates for splenectomy or as bridging therapy before splenectomy [18]. Other immunosuppressive agents, such as azathioprine, cyclosporine, and cyclophosphamide, were previously administered after failure of glucocorticoids, rituximab, and splenectomy [13]. However, because of their limited efficacy and substantial toxicity, they have gradually been replaced by targeted therapies with the development of novel agents (Table 2).

**Table 2:** Research stages of novel agents for the treatment of wAIHA

Mechanism		Representative drug	Phase	Efficiency
Complement inhibitors	C3	Pegcetacoplan	Phase 2/3	Hemoglobin plateau at week 8; 64% transfusion-independent; reduction in hemolysis markers
	Factor B	Iptacopan	Phase 1	Trial ongoing (NCT06847607)
Plasma cell -targeted	CD38 monoclonal antibody	Daratumumab	Case report; retrospective study	50% CR (Hb > 120 g/L); median remission 5.5 months; effective in rituximab-refractory cases
	Proteasome inhibitor	bortezomib	Phase 2	With rituximab: 88% ORR, 56.7% CR; with dexamethasone: 75% remission rate; median time to response 1.8 months
**B cell targeted**	PI3Kδ inhibitor	Parsaclisib	Phase 2	52% Hb increase at week 1; 75% ORR, 50% CR at 12 weeks; steroid-sparing effect
	CAR-T cell	Autologous CD19 CAR-T cell	Phase 1	91% and 100% ORR at 1 and 3 months, respectively; median drug-free remission 11.5 months; low-grade CRS only
	BTK inhibitor	Ibrutinib	Case report	2/2 PR at 2 weeks; CR at 6–10 weeks
		Orelabrutinib	Phase 1	9/12 responded; 3/3 CR at 3 months, sustained at 5–8 months
		Rilzabrutinib	Phase 2	64% ORR; 41% sustained response
		Zanubrutinib	Phase 2	44.4% ORR, CR in 2/9 and PR in 2/9
	mTOR inhibitor	Sirolimus	Retrospective study	79.5% ORR; 56.8% CR; median time to response 2 months; median relapse-free survival 19 months
	BAFF	Ianalumab	Phase 2/3	Trials ongoing (NCT05648968; NCT07039422)
**FcRn**		Nipocalimab	Phase 2/3	Trials ongoing; rapid clearance of pathogenic IgG
**Syk inhibitor**		Fostamatinib	Phase 3	Phase 2: 46% achieved the primary endpoint; 63% had Hb increase ≥ 1.5 g/dL. FORWARD Phase 3: 35.6% response rate vs. 26.7% with placebo
		Sovleplenib	Phase 2/3	67% ORR at week 24; 48% sustained remission; median time to response 1.3 weeks; 95.2% transfusion-free

Abbreviations: BAFF, B-cell activating factor; BCMA, B-cell maturation antigen; BTK, Bruton tyrosine kinase; CAR-T, chimeric antigen receptor T-cell; CR, complete remission; CRS, cytokine release syndrome; FcRn, neonatal Fc receptor; Hb, hemoglobin; Ig, immunoglobulin; LDH, lactate dehydrogenase; mTOR, mechanistic target of rapamycin; ORR, overall response rate; PI3Kδ, phosphoinositide 3-kinase delta; PNH, paroxysmal nocturnal hemoglobinuria; PR, partial remission; Syk, spleen tyrosine kinase; wAIHA, warm autoimmune hemolytic anemia

### Complement inhibitor

Given the critical role of autoantibody-mediated RBC destruction through activation of the classical complement pathway in the pathogenesis of AIHA, complement inhibitors represent a novel therapeutic strategy and have demonstrated promising clinical efficacy [19].

Pegcetacoplan is a subcutaneously administered C3-targeted therapy that has shown preliminary efficacy in the treatment of wAIHA. In a clinical trial conducted by Eloy et al. (NCT03226678), 11 patients with wAIHA who were unresponsive to glucocorticoids or rituximab, or who relapsed after treatment, received pegcetacoplan. Ten patients experienced treatment-related adverse events, primarily diarrhea, headache, hypertension, nausea, and vitamin B12 deficiency, all of which were mild to moderate in severity. After treatment, the median hemoglobin concentration increased steadily and reached a plateau at week 8. Lactate dehydrogenase (LDH), indirect bilirubin, and reticulocyte counts were significantly reduced, whereas direct antiglobulin test positivity increased. During the treatment period, 64% of patients remained transfusion-independent, and fatigue scores improved in all patients [20]. Based on the favorable safety and efficacy observed in the Phase II study, a Phase III placebo-controlled trial (NCT05096403) is currently underway.

Unlike pegcetacoplan, iptacopan is an upstream factor B inhibitor of the complement pathway. It specifically binds to factor B, thereby inhibiting the formation of C3 convertase and blocking amplification loop–mediated complement activation. Through this mechanism, iptacopan controls both terminal C5-mediated intravascular hemolysis and proximal C3-mediated extravascular hemolysis by preventing downstream effector generation and terminal pathway amplification, ultimately achieving effective control of hemolysis [21, 22]. Iptacopan has been approved for the treatment of paroxysmal nocturnal hemoglobinuria, and a clinical trial evaluating iptacopan in wAIHA (NCT06847607) is ongoing. The results of this study are awaited.

### B cell targeted therapies

#### mTOR pathway inhibitors

The PI3K/AKT/mTOR signaling pathway is a key regulator of cell survival, proliferation, differentiation, metabolism, and apoptosis. This axis plays a critical role in maintaining B-cell homeostasis and regulating immune responses. Sirolimus is a macrolide immunosuppressant that modulates immune cell metabolism and function by inhibiting mTOR. It suppresses proinflammatory Th1 and, Th17 cells, as well as M1 macrophages, while promoting anti-inflammatory regulatory T cells and M2 macrophages, thereby exerting therapeutic effects in autoimmune diseases [23]. In a single-center retrospective study by Zhang et al., 44 patients with relapsed or refractory wAIHA and Evans syndrome (including 37 patients with wAIHA) received sirolimus. An overall remission rate of 79.5% was observed, with 56.8% achieving complete remission and a median time to response of 2 months. The overall response rates at 12 months and at the end of follow-up were 79.5% and 72.7%, respectively, with a complete response rate of 50.0% at both time points. Five patients experienced relapse, with a median time to relapse of 19 months. The most common adverse events during treatment were oral ulcers, infections, and elevated transaminase levels [24]. Overall, sirolimus demonstrated a high remission rate, a relatively low relapse rate, and manageable adverse effects in wAIHA, suggesting that it may represent a potential therapeutic option for relapsed or refractory disease. A prospective Phase II trial (NCT05925023) is currently underway.

### BTK inhibitors

BTK inhibitors suppress autoreactive B-cell function and reduce autoantibody production by blocking BTK, a key molecule in the B-cell receptor signaling pathway, thereby mitigating erythrocyte destruction in wAIHA. In addition, BTK is a critical kinase in the Fc receptor signaling cascade, and BTK inhibitors can inhibit macrophage-mediated phagocytosis and destruction of sensitized RBCs via Fc receptors [25]. BTK inhibitors first demonstrated therapeutic potential in wAIHA among patients with chronic lymphocytic leukemia. Since then, several studies have evaluated their use as monotherapy in wAIHA. In case reports, two patients achieved transfusion independence and partial remission within two weeks of treatment with the first-generation BTK inhibitor ibrutinib and subsequently achieved complete remission with compensated hemolysis at six and ten weeks, respectively [26]. As a second-generation BTK inhibitor, orelabrutinib has shown preliminary efficacy in wAIHA; among 12 patients with refractory wAIHA or Evans syndrome enrolled in one study, nine achieved a clinical response.

Among these patients, three who were followed for six months achieved complete remission within 2–3 weeks of oral orelabrutinib administration, with sustained efficacy during 5–8 months of follow-up; only one patient experienced reversible myelosuppression [27]. Preliminary results have also been reported for the third-generation BTK inhibitor rilzabrutinib in the treatment of wAIHA (NCT05002777). Twenty-one patients with primary wAIHA received oral rilzabrutinib for 24 weeks, and responders entered a long-term extension study. The overall hemoglobin response rate was 64%, of which 41% represented sustained responses [28]. In addition, zanubrutinib has demonstrated preliminary efficacy and manageable safety in a prospective, single-arm Phase II study in wAIHA, providing supportive data for future randomized controlled trials [29]. Furthermore, clinical trials investigating the second-generation BTK inhibitor acalabrutinib in patients with AIHA and concomitant chronic lymphocytic leukemia (NCT04657094) are ongoing. In summary, BTK inhibitors represent a novel therapeutic strategy targeting the B-cell signaling pathway and have shown encouraging potential in AIHA. They may become an important treatment option for relapsed or refractory AIHA in the future.

### PI3Kδ inhibitors

Phosphoinositide 3-kinase delta (PI3Kδ), a member of the class I phosphatidylinositol 3-kinase family, plays a critical role in regulating key cellular processes, including proliferation, survival, growth, and metabolism. Dysregulation of PI3Kδ signaling disrupts central and peripheral B-cell tolerance, impairs the induction of B-cell anergy, and synergizes with T follicular helper cell expansion and regulatory T-cell dysfunction, thereby promoting autoreactive B-cell activation and autoantibody production. PI3Kδ inhibitors have demonstrated therapeutic potential in autoimmune diseases [30, 31].

Parsaclisib is a highly selective second-generation PI3Kδ inhibitor. In a Phase II study (NCT03538041), 52% of 16 patients with previously treated wAIHA experienced a hemoglobin increase during the first week of oral parsaclisib administration. The overall response rate reached 75% at 12 weeks, with 50% of patients achieving complete response. Among the 11 patients who were prednisone-dependent at baseline, five discontinued prednisone completely and reported improved quality of life [32]. These findings suggest that parsaclisib has a rapid onset of action and sustained therapeutic benefit, providing a potentially safe and effective option for refractory wAIHA. Additional clinical trials evaluating PI3Kδ inhibitors in wAIHA are ongoing.

### BAFF pathway inhibitors

BAFF is a cytokine belonging to the tumor necrosis factor ligand family and plays a critical role in B-cell development, maturation, and survival. In wAIHA, BAFF overexpression is associated with disruption of B-cell tolerance and increased autoantibody production [6]. BAFF binds to its receptor, BAFF receptor (BAFF-R), enhancing the expression of antiapoptotic proteins of the BCL-2 family, inhibiting the mitochondrial apoptotic pathway, and rescuing mature self-reactive B cells that have escaped negative selection. This process prolongs their survival and expands the peripheral autoreactive B-cell pool, thereby providing a cellular source for sustained autoantibody production [33]. Based on the successyaful clinical application of belimumab in autoimmune diseases such as systemic lupus erythematosus and ITP, targeting the BAFF pathway may offer a promising therapeutic strategy for wAIHA [34]. Currently, clinical trials investigating the BAFF-R inhibitor ianalumab (NCT05648968, NCT07039422) in wAIHA are ongoing.

### CAR-T cell

Chimeric antigen receptor T-cell (CAR-T) therapy has been widely used in the treatment of hematologic malignancies. In autoimmune diseases, CAR-T cells targeting B-cell–specific surface antigens, such as CD19 and CD20, can induce rapid and sustained depletion of circulating B cells, thereby promoting disease remission [35]. Based on this mechanism, CAR-T cell therapy has been applied in autoimmune diseases, including systemic lupus erythematosus, multiple sclerosis, and myasthenia gravis, with encouraging therapeutic outcomes. The safety and efficacy of CAR-T therapy in AIHA are currently under investigation [36]. In a study by Li et al., 11 patients with relapsed wAIHA received CD19-targeted CAR-T cell therapy following lymphodepleting preconditioning with fludarabine and cyclophosphamide. The median follow-up duration was 12.2 months. The overall remission rates at 1 and 3 months after infusion were 91% and 100%, respectively. The median duration of drug-free remission was 11.5 months. Nine patients developed low-grade cytokine release syndrome (CRS), and one patient developed immune effector cell–associated neurotoxicity syndrome (ICANS); no grade ≥ 3 CRS or ICANS events were reported [37, 38]. CD19-targeted CAR-T cells induce immune reconstitution through profound B-cell depletion, demonstrating rapid, deep, and sustained remission in patients with wAIHA and an acceptable safety profile. These findings support CAR-T therapy as a potential salvage treatment option for wAIHA. Moreover, this study provides a rationale for larger-scale clinical trials in the future.

### Plasma cell–targeted therapies

#### Proteasome inhibitors

Bortezomib is a proteasome inhibitor widely used in the treatment of multiple myeloma and has also demonstrated therapeutic potential in AIHA. By downregulating the expression of various proinflammatory cytokines and antiapoptotic molecules, selectively depleting long-lived plasma cells that are resistant to conventional therapies, and attenuating the antigen-presenting capacity of dendritic cells and macrophages, bortezomib exerts multitarget, rapid, and sustained immunomodulatory effects in autoimmune diseases [39, 40].

Fadlallah et al. retrospectively analyzed eight patients with relapsed or refractory wAIHA who received bortezomib combined with dexamethasone. After six treatment cycles, six patients achieved disease remission, including three who achieved complete remission. After a median follow-up of 14 months, sustained clinical improvement was observed in these six patients. Five patients experienced moderate treatment-related adverse events, and no treatment-related deaths occurred [41]. These preliminary findings suggest that bortezomib combined with dexamethasone may represent a promising and relatively safe therapeutic strategy for patients with multirefractory wAIHA; however, prospective clinical trials are required to further evaluate its efficacy and safety. In a study by Neri et al., a patient with relapsed or refractory IgA-mediated wAIHA achieved transfusion independence and discontinued all medications after two cycles of bortezomib combined with glucocorticoids, attaining complete remission that was sustained for three years [42].

In theory, combination therapy with bortezomib and rituximab can simultaneously deplete plasma cells and B cells, thereby enhancing therapeutic efficacy in AIHA. In a Phase II clinical study, 30 patients with relapsed or refractory wAIHA received this regimen. The overall response rate at 6 months—the primary endpoint—was 88%, with a complete response rate of 56.7%. After a median follow-up of 22 months, the final ORR was 80%, and the complete remission rate was 63.3%. Treatment-related adverse events occurred in 60% of patients, most commonly pulmonary infections, fever, and thrombocytopenia; 23.3% were grade ≥ 3. Immune monitoring showed sustained B-cell depletion, a reduced proportion of regulatory T cells, and no significant reversal of CD8 + T-cell activation [43]. Chen et al. also administered bortezomib combined with rituximab to seven patients with wAIHA, achieving an ORR of 100%. After a short treatment course, four patients relapsed; among them, two achieved long-term remission after receiving rituximab monotherapy as maintenance therapy following a second course of combination treatment [44]. These studies suggest that the combination of bortezomib and rituximab can rapidly achieve disease control and significantly reduce dependence on glucocorticoids. However, a single treatment course appears insufficient to maintain long-term remission, and further investigation of repeated courses or rituximab maintenance therapy is warranted. Overall tolerability was acceptable, although careful monitoring for infectious complications is necessary.

In a single-center study, two patients with refractory wAIHA received bortezomib, three received bortezomib combined with daratumumab, and one received daratumumab monotherapy. Ultimately, five patients achieved hematologic remission, including four cases of complete remission and one case of partial response [45]. The median time to response with bortezomib with or without daratumumab, targeting long-lived plasma cells, was 1.8 months after treatment initiation, supporting the inclusion of plasma cell–targeted therapy as a salvage treatment option for refractory wAIHA. However, prospective studies are required to determine the optimal dosage, treatment duration, and sequencing strategies in combination with existing therapies.

Bortezomib induces plasma cell apoptosis and inhibits inflammatory signaling through multiple mechanisms, thereby exerting immunosuppressive effects and representing a potential therapeutic approach for wAIHA. Nevertheless, current studies involve small sample sizes, and large-scale randomized controlled trials are lacking to validate the efficacy of monotherapy or combination therapy with monoclonal antibodies in relapsed or refractory wAIHA.

### Monoclonal antibodies

CD38 is a surface antigen widely expressed on normal plasma cells and multiple myeloma cells and represents a well-established therapeutic target in myeloma. Daratumumab is a monoclonal antibody directed against CD38. In ITP, daratumumab exerts cytotoxic effects on CD38-positive plasma cells through multiple mechanisms, including complement-dependent cytotoxicity, antibody-dependent cellular cytotoxicity, and antibody-dependent cellular phagocytosis, thereby reducing autoantibody production. In addition, daratumumab modulates the overactivated immune microenvironment, facilitating the restoration of immune tolerance to platelet autoantigens [46]. Consequently, daratumumab has emerged as a second-line salvage therapy for ITP [47]. Based on its favorable safety and efficacy profile in ITP, the therapeutic potential of daratumumab in autoantibody-mediated AIHA is currently under investigation. Marit et al. conducted a retrospective analysis of 12 patients with severe refractory wAIHA who received daratumumab. Complete remission was achieved in 50% of patients, defined as a hemoglobin level > 120 g/L, with a median remission duration of 5.5 months [48]. In a report by Yaqeen et al., a patient with aplastic anemia developed refractory hemolytic anemia after hematopoietic stem cell transplantation. Following four weeks of daratumumab monotherapy, hemoglobin levels improved significantly and normalized by week 8. Hemolytic markers and autoantibodies became undetectable during the subsequent six-month follow-up, without the need for additional glucocorticoid or immunosuppressive therapy [49]. In the report by Max J et al., four patients with wAIHA refractory to rituximab received daratumumab. During follow-up, two patients achieved sustained disease control, whereas the remaining two required alternative therapy due to clinical relapse [50]. Catharina et al. also described three patients with severe post-transplant AIHA who received daratumumab. Ultimately, two patients achieved remission, whereas one experienced relapse eight months after treatment [51]. Collectively, existing evidence provides preliminary support for the therapeutic potential of daratumumab in wAIHA. However, large-scale randomized controlled trials are still lacking to confirm its efficacy and define its role in the management of wAIHA, and further investigation is warranted.

### BCMA-targeted therapy

Although CD19-targeted CAR-T cells have demonstrated feasibility in current studies, some patients experience relapse within several months after treatment. With the expanding application of CAR-T therapy in autoimmune diseases, evaluating long-term efficacy and elucidating mechanisms of relapse have become critical challenges. Further investigation by Li et al. revealed that interactions among HLA-DRB5^+^ B cells, activated CD4^+^ T cells, and long-lived B cells expressing B-cell maturation antigen (BCMA) contribute to the formation of a relapse-associated B-cell microenvironment, resulting in sustained autoantibody production and disease recurrence [38]. Given the expression of BCMA on memory B cells and plasma cells, BCMA-targeted therapy has emerged as a promising therapeutic strategy for wAIHA. Zhang et al. reported two patients with AIHA who received bispecific antibody therapy targeting BCMA and CD3, including one patient with Evans syndrome and concomitant ITP; both had relapsed after CD19-targeted CAR-T therapy. Following bispecific antibody treatment, peripheral plasma cells and memory B cells markedly decreased in both patients, hemolysis was rapidly controlled, and hemoglobin levels normalized. At six months of follow-up, both patients remained in sustained remission [52]. In another study by Li et al., two patients relapsed 7.3 and 8.5 months after CD19-targeted CAR-T therapy and subsequently achieved sustained remission after salvage treatment with BCMA- and CD3-targeted bispecific antibodies [38]. These findings suggest that BCMA- and CD3-directed bispecific antibodies may represent an effective rescue strategy for relapse after CD19-targeted CAR-T therapy in wAIHA. Furthermore, these observations provide additional clinical evidence supporting the contribution of plasma cells to autoimmune disease relapse.

### Inhibitions of phagocytosis

#### Syk inhibitor

Spleen tyrosine kinase (Syk) is an intracellular tyrosine kinase primarily expressed in hematopoietic and bone marrow–derived cells and is essential for B-cell development, maturation, and antibody production. Aberrantly increased expression or sustained phosphorylation of Syk amplifies autoantigen-mediated immune injury, making it a key therapeutic target for interrupting the pathogenic cascade and restoring immune homeostasis [53]. In AIHA, Syk inhibitors reduce autoantibody production and inhibit tyrosine kinase–dependent Fcγ receptor–mediated macrophage phagocytosis, thereby decreasing RBC destruction. This approach represents an effective therapeutic strategy for wAIHA [54].

Fostamatinib is a spleen tyrosine kinase (Syk) inhibitor whose active metabolite (R406) blocks Fc receptor– and B-cell receptor–mediated signaling. It is approved for adult chronic immune thrombocytopenia and has been evaluated in wAIHA based on its ability to inhibit autoantibody-driven cytopenias. In a Phase II clinical study of 24 heavily pretreated patients with baseline hemoglobin < 10 g/dL, fostamatinib demonstrated clinically meaningful activity, with 46% of patients achieving the primary endpoint (hemoglobin > 10 g/dL with an increase ≥ 2 g/dL) and 63% showing a hemoglobin increase ≥ 1.5 g/dL. Hematologic improvement was often early and accompanied by reduced hemolysis markers (LDH and reticulocyte counts). Treatment was generally tolerable, with mainly mild-to-moderate adverse events such as diarrhea, fatigue, and hypertension [55]. In the subsequent Phase III FORWARD trial, fostamatinib did not significantly improve the primary response rate compared with placebo overall (35.6% vs. 26.7%), although subgroup analyses suggested potential benefit in selected geographic populations [56].

Overall, fostamatinib represents the first oral targeted Syk inhibitor to demonstrate clinical activity in wAIHA, with a mechanism that simultaneously modulates autoantibody production and Fc receptor–mediated phagocytosis. Although its Phase III results were not definitively positive, it remains a promising mechanism-based option for relapsed or refractory wAIHA and highlights the therapeutic potential of Syk pathway inhibition.

Sovleplenib is a novel, selective, orally administered small-molecule Syk inhibitor that has demonstrated preliminary efficacy and safety in the treatment of wAIHA in a Phase II/III clinical trial (NCT05535933). In this randomized study, sovleplenib demonstrated a rapid onset of action, with hemoglobin improvement observed within approximately 1–2 weeks. By week 24, 67% of patients achieved a hemoglobin response, and nearly half achieved sustained remission. A high proportion of patients (95.2%) remained transfusion-independent during treatment, indicating durable clinical benefit. Sovleplenib was generally well tolerated. No grade ≥ 4 adverse events were reported, and the most common severe adverse event was anemia unrelated to treatment. Overall, sovleplenib showed rapid and clinically meaningful hematologic improvement with an acceptable safety profile, supporting its potential as a targeted Syk inhibitor for wAIHA [57].

### FcRn inhibitors

The neonatal Fc receptor (FcRn) is a transmembrane protein originally identified in neonatal intestinal epithelial cells. It binds to the Fc fragment of IgG in pH-dependent manner, protecting IgG from lysosomal degradation and recycling it back into the circulation, thereby prolonging its half-life [58]. In AIHA, FcRn functions as an amplifier of extravascular hemolysis by recycling pathogenic IgG, extending its half-life and promoting sustained destruction of RBCs. FcRn inhibitors competitively block the interaction between IgG and FcRn in vivo, thereby accelerating IgG clearance and representing a promising therapeutic strategy for wAIHA [59]. Currently, clinical trials investigating the FcRn inhibitor nipocalimab and other related agents in wAIHA are ongoing, offering potential new treatment options for patients with refractory disease.

### Other emerging targets

Zhao et al. investigated the role of IL-6 in wAIHA by analyzing plasma samples from affected patients. The results showed that plasma IL-6 levels during hemolytic episodes were significantly higher than those in healthy controls and were negatively correlated with hemoglobin levels and positively correlated with LDH levels. IL-6 levels were also significantly higher in glucocorticoid-resistant patients than in treatment-responsive patients. In vitro stimulation with IL-6 significantly promoted B-cell activation while suppressing IL-10 secretion [60]. Based on these findings, the IL-6 receptor antagonist tocilizumab may inhibit excessive B-cell activation by blocking IL-6 signaling and is being explored as a potential targeted therapy for glucocorticoid-resistant wAIHA [61].

In short, for wAIHA, glucocorticoids remain the first-line treatment. Rituximab has replaced splenectomy as the preferred second-line therapy because of its favorable response rate, although its onset of action may be delayed. Novel B-cell–targeted agents, including BTK inhibitors, PI3Kδ inhibitors, and BAFF inhibitors, have demonstrated promising efficacy. In addition, Syk inhibitors and FcRn inhibitors, which interfere with antibody-mediated phagocytosis and IgG recycling, provide alternative options for glucocorticoid-resistant disease. Furthermore, CD38-targeted monoclonal antibodies and proteasome inhibitors directed at plasma cells, as well as complement inhibitors and CD19-targeted CAR-T cell therapy, offer additional therapeutic avenues for refractory wAIHA.

### cAIHA

Cold agglutinin–mediated cAIHA accounts for 25%–30% of all AIHA cases. CAD is a clonal B-cell lymphoproliferative disorder characterized by abnormal B-cell clones and activation of the classical complement pathway and is often associated with infections or malignant tumors. Clonal B cells in the bone marrow continuously produce cold-reactive autoantibodies, predominantly monoclonal IgM κ, directed against RBC antigens. These antibodies bind to RBCs at low temperatures and activate the classical complement pathway, thereby inducing complement-dependent hemolysis [62, 63]. Unlike wAIHA, conventional glucocorticoid therapy has limited efficacy in cAIHA. In contrast, B-cell–targeted therapies and complement inhibitors have demonstrated favorable efficacy in cAIHA (Table 3).

**Table 3:** The research stage and achievements of new drugs in the treatment of cAIHA

Mechanism		Representative drug	Phase	Outcomes
**Complement inhibitors**	C1s	Riliprubart	Phase II	Hemoglobin plateau by day 29; complement levels normalized; sustained response for 15 weeks; well tolerated
	C1q	C-INH	Phase II	7/10 achieved the primary endpoint; reduced C3d deposition; limited effect on hemolysis markers
	C1s	Sutimlimab	Phase III	73% vs. 15% achieved Hb increase ≥ 15 g/L; mean Hb increase 27 g/L; sustained efficacy up to 3 years; improved fatigue
	C3	Pegcetacoplan	Phase III	Rapid Hb increase; 54% transfusion-independent at 12 months
	C5	Eculizumab	Phase II	Reduced LDH; improved intravascular hemolysis; no significant effect on extravascular hemolysis or cold-induced symptoms
	Factor B	Iptacopan	Case report	Rapid achievement of transfusion independence; sustained remission at 4 months
**Plasma cell–targeted**	CD38 monoclonal antibody	Daratumumab	Case report	Hb increase at 2 weeks; 3/7 CR and 1/7 PR; sustained CR > 2 years in one case; improved cold symptoms
	Proteasome inhibitor	Bortezomib	Case report	31.6% ORR and 15.8% CR in relapsed CAD; DVd regimen achieved sustained CR in refractory CAS
**B-cell–targeted**	CD19 and FcγRIIb inhibitor	Obexelimab	Phase II	Trial ongoing
	PI3Kδ inhibitor	Parsaclisib	Phase II	50% ORR; limited efficacy (only 1 CR)
	BTK inhibitor	Ibrutinib	Case report	12/13 CR and 1/13 PR; improved acrocyanosis (9/9); median duration 12 months
	CAR-T cell	Autologous CD19-targeted CAR-T cell	Case report	Complete and durable remission; normalized Hb; negative cold agglutinins

Abbreviations: BTK, Bruton tyrosine kinase; CAD, cold agglutinin disease; CAR-T, chimeric antigen receptor T-cell; CAS, cold agglutinin syndrome; cAIHA, cold autoimmune hemolytic anemia; C-INH, C1 inhibitor; CR, complete remission; DVd, daratumumab, bortezomib, and dexamethasone; Hb, hemoglobin; Ig, immunoglobulin; IgM, immunoglobulin M; LDH, lactate dehydrogenase; ORR, overall response rate; PI3K, phosphoinositide 3-kinase; PR, partial remission

### Frontline treatment

Rituximab remains the cornerstone of therapy for cAIHA, and its use has evolved from monotherapy to combination regimens, resulting in improved clinical outcomes with optimized treatment strategies [62]. The addition of oral fludarabine achieved a remission rate of 76%, with a complete remission rate of 21% and a median remission duration exceeding 66 months. However, this regimen is associated with substantial bone marrow toxicity and limited tolerability in older patients [64]. The combination of rituximab and bendamustine (BR) has become a first-line treatment option for patients with severe cAIHA. A multicenter study conducted in Northern Europe demonstrated an overall response rate of 71% with BR therapy, including 40% complete remission and a median remission duration of 32 months [65]. However, rituximab shows limited efficacy in patients with prominent plasma cell differentiation and does not eliminate long-lived plasma cells, which may contribute to disease recurrence in some cases [66].

### Complement inhibitors

Riliprubart is a humanized monoclonal antibody that selectively inhibits activated complement component C1s. In a study by Shirley et al., the safety and efficacy of riliprubart in CAD were evaluated for the first time. A total of 12 patients who completed the full course of riliprubart were included in the final analysis. The treatment demonstrated a favorable safety profile, with most adverse events being grade 1–2 headaches and arthralgia. No treatment-related serious adverse events, deaths, treatment discontinuations, or infection or thrombosis events were reported. Hemoglobin levels gradually increased and reached a plateau by day 29; bilirubin levels rapidly decreased, and complement levels normalized, with these improvements sustained for 15 weeks [67]. These findings indicate that rapid and sustained inhibition of C1s can effectively control hemolysis and improve anemia.

Esther et al. conducted a clinical trial evaluating complement C1q inhibition in cAIHA. This study included 10 patients with severe cAIHA who received four doses of the C1q inhibitor C1 inhibitor (C1-INH) administered before transfusion and within 36 h thereafter. The results showed that seven patients met the primary endpoint, defined as the absence of a hemoglobin decrease of ≥ 10 g/L within 24 h after transfusion. However, retrospective analysis revealed that six patients had already demonstrated stable hemoglobin levels during prior transfusion cycles without C1-INH before enrollment, indicating limited sensitivity of this endpoint. Following C1-INH treatment, C3d deposition on the surface of RBCs significantly decreased; however, hemolysis-related parameters, including LDH and total bilirubin, did not improve. No significant changes were observed in systemic complement activation markers or inflammatory indices, and transfusion requirement were not reduced. Regarding safety, no serious treatment-related adverse events were reported [68]. Although C1-INH transiently inhibited complement deposition on RBCs at the administered dose, it did not significantly suppress hemolysis, reduce transfusion requirements, or attenuate systemic complement or inflammatory activation. Therefore, this regimen is not supported as routine acute-phase therapy for severe cAIHA. Future studies should explore higher doses, extended treatment durations, or alternative complement-targeted agents.

Sutimlimab is a humanized IgG4 monoclonal antibody that selectively inhibits C1s, thereby blocking classical complement activation while preserving the alternative and lectin pathways. Early-phase studies demonstrated rapid and sustained increases in hemoglobin, reduced transfusion requirements, and acceptable long-term safety in patients with CAD [69]. In a 26-week randomized placebo-controlled trial, 73% of patients receiving sutimlimab achieved a hemoglobin increase of ≥ 15 g/L, compared with 15% in the placebo group [70, 71]. Sutimlimab also significantly improved hemolysis markers, fatigue, and quality of life. Adverse events were predominantly mild to moderate, with no treatment-related deaths reported [72]. Overall, the C1s inhibitor sutimlimab rapidly blocks the classical complement pathway, provides sustained control of hemolysis, and improves quality of life. It has become a first-line targeted biologic therapy for both transfusion-dependent and non–transfusion-dependent CAD and has been approved for the treatment of CAD in multiple countries.

Pegcetacoplan has also demonstrated favorable efficacy in cAIHA. In the study by Eloy et al., an additional cohort of 13 patients with CAD was included. The median hemoglobin level increased within the first four weeks of pegcetacoplan treatment and remained stable thereafter. Hemolysis-related parameters, including LDH, indirect bilirubin, and reticulocyte count, significantly decreased, accompanied by an increase in hemoglobin levels and improvement in fatigue scores. Furthermore, 54% of patients remained transfusion-independent at 12 months [20]. These findings indicate that C3 inhibition can rapidly and sustainably suppress complement-mediated hemolysis and improve quality of life, supporting the therapeutic potential of C3 inhibitors in cAIHA.

Eculizumab is a complement C5 inhibitor approved for the treatment of paroxysmal nocturnal hemoglobinuria. As a terminal complement inhibitor, it effectively suppresses intravascular hemolysis but does not prevent C3b-mediated extravascular hemolysis, thereby limiting its clinical applicability. Early data from the DECADE study evaluated the safety and efficacy of eculizumab in cAIHA. Thirteen patients were treated, including 12 with CAD and 1 with CAS. The results demonstrated that eculizumab significantly reduced LDH levels and improved anemia; however, it did not improve markers of extravascular hemolysis, such as C3b deposition, or cold-induced circulatory symptoms [73]. These findings indicate that although terminal complement inhibition effectively controls intravascular hemolysis and reduces transfusion requirements, it does not block extravascular hemolysis or IgM-mediated red blood cell agglutination and requires continuous administration to maintain efficacy. Therefore, it may be more appropriate as an alternative option for patients who are refractory to or intolerant of B-cell–targeted therapy.

Iptacopan inhibits complement factor B and prevents the formation of C3 convertase, thereby suppressing both intravascular and extravascular hemolysis. In a case report by Fang et al., a patient with CAD secondary to lymphoplasmacytic lymphoma was intolerant to rituximab combined with bendamustine and exhibited progressive hemolysis despite subsequent treatment with zanubrutinib. After initiation of oral iptacopan combined with cyclophosphamide and dexamethasone, the patient achieved transfusion independence, recovery of hemoglobin levels, and normalization of bilirubin and LDH, with sustained remission during a four-month follow-up period [74]. This case suggests that iptacopan may rapidly ameliorate hemolysis in CAD, and warrants further investigation in larger-scale studies.

In summary, given the central role of the classical complement pathway in the pathogenesis of cAIHA, complement inhibitors represent a major therapeutic advance in its management. Future research should focus on combination strategies integrating complement inhibition with B-cell– or plasma cell–targeted therapies to achieve personalized and precision-based treatment approaches.

### Plasma cell–targeted therapy

Daratumumab has shown potential feasibility in the treatment of cAIHA. Oliver et al. first reported the use of daratumumab for CAD. The patient initiated daratumumab after failure of glucocorticoid and rituximab therapy. Within two weeks, hemoglobin levels increased, followed by normalization of LDH and bilirubin. Serum IgM levels and cold agglutinin titers decreased, and clinical symptoms significantly improved [75]. Marit et al. also included seven patients with CAD in their study. Three patients achieved complete remission after treatment, and one patient achieved sustained partial remission after 12 weeks. Six patients with acrocyanosis experienced improvement in peripheral cyanosis, and four patients reported symptomatic improvement [48]. In a case report by Anna et al., a patient with CAD achieved complete remission lasting more than two years after daratumumab treatment. Further cytokine analysis demonstrated downregulation of tumor necrosis factor-α, biphasic modulation of interferon-γ, and upregulation of IL-6, IL-10, IL-17, and transforming growth factor-β, further supporting the immunomodulatory effects of daratumumab [76]. As a monoclonal antibody targeting CD38, daratumumab reduces hemolysis and effectively improves cold-induced circulatory symptoms by depleting CD38-positive cells and modulating the immune microenvironment. It represents a potential therapeutic option for the future management of CAD.

In the early GIMEMA study, 20 patients with CAD who relapsed after first-line therapy received four cycles of bortezomib. Among the 19 evaluable patients, the overall response rate was 31.6%, including a complete remission rate of 15.8%. After a median follow-up of 16 months, 66.7% of responding patients maintained remission. One patient who relapsed achieved remission again after re-administration of bortezomib [77]. This study provides preliminary evidence supporting the use of bortezomib as salvage therapy for patients with CAD who lack effective second-line treatment options. Further research is warranted to evaluate extended treatment courses or repeated administration of bortezomib monotherapy, as well as combination regimens of bortezomib and rituximab in CAD.

In the study by McGlothlin et al., two patients with glucocorticoid-refractory CAD were treated with bortezomib and daratumumab, respectively, and both achieved complete remission with sustained responses at two months, suggesting that plasma cell–targeted therapy may represent a salvage option for CAD. However, these findings require validation in larger clinical studies [45]. Rybinski et al. reported a patient with severe CAS who was unresponsive to rituximab and BTK inhibitors and was subsequently treated with bortezomib combined with daratumumab. After bridging therapy with sutimlimab, bone marrow examination revealed clonal IgM-secreting plasma cells. The patient then received daratumumab, bortezomib, and dexamethasone (DVd), while sutimlimab was continued during treatment. Ultimately, the patient achieved sustained complete remission off therapy, without evidence of hemolysis, relapse, or detectable monoclonal protein [78]. This study provides preliminary evidence that a short-term DVd regimen may induce durable remission in selected cases of refractory CAS, particularly in patients with IgM-positive plasma cell clones, and that sutimlimab may serve as an effective bridging therapy. This strategy introduces a potential treatment pathway aimed at achieving treatment-free remission and targeting the underlying plasma cell clone in cAIHA.

Agents such as daratumumab and bortezomib that target IgM-secreting plasma cells may offer a potential curative approach for patients who are refractory to rituximab and exhibit plasma cell clonality. This approach represents an important direction toward achieving functional cure in the future.

### B-cell–targeted therapy

Obexelimab is a bifunctional humanized monoclonal antibody that binds to CD19 and FcγRIIb, thereby inhibiting B-cell activation without inducing B-cell depletion. In vitro studies have demonstrated that obexelimab directly suppresses the production of pathogenic red blood cell autoantibodies, with limited effects on cytokine modulation [79]. These findings suggest that obexelimab exerts its effects primarily through targeted B-cell inhibition rather than broad immunosuppression, providing a mechanistic rationale for its development as a novel non–B-cell–depleting therapy for relapsed and refractory disease. Its efficacy and safety are currently being evaluated in a Phase II clinical trial (NCT05786573).

The clinical study of parsaclisib, a PI3K δ inhibitor, also included patients with CAD. The overall response rate in patients with CAD was 50%, with only one patient achieving complete remission of 49% [32]. Although parsaclisib demonstrated activity in CAD, its efficacy appeared limited, possibly owing to the relatively low burden of pathogenic B-cell clones in cAIHA. Future studies should explore combination strategies with complement inhibitors or other targeted therapies.

BTK inhibitors have shown promising efficacy in wAIHA; however, data in cAIHA remain limited. In a study by Cooper et al., 13 patients with cAIHA received ibrutinib monotherapy and demonstrated substantial clinical benefit: 12 achieved complete remission, one achieved partial remission, and nine patients with acrocyanosis experienced symptomatic improvement. The median treatment duration was 12 months [80]. These findings indicate that BTK inhibition may rapidly and durably improve hemolysis and peripheral cyanosis in cAIHA, providing a valuable therapeutic option for patients who are refractory to rituximab or complement inhibitors.

Currently, research on CAR-T therapy in cAIHA remains limited. Ding et al. reported the use of CAR-T therapy in a patient with lymphoma-associated CAS. After being diagnosed with diffuse large B-cell lymphoma, the patient developed secondary CAS characterized by peripheral cyanosis. Following failure of multiple lines of therapy, the patient received autologous CD19-targeted CAR-T cell infusion. Hemoglobin levels returned to normal, hemolysis-related parameters became negative, cold agglutinin titers remained undetectable, and CAS symptoms did not recur [81]. CAR-T cells may achieve deep disease control by eradicating malignant B cells—the source of pathogenic cold agglutinins—while also depleting normal B cells, thereby reducing autoantibody production. This case suggests that CAR-T therapy may represent a potential curative strategy for lymphoma-associated CAS.

### Conclusion and future perspectives

AIHA, a red blood cell–destructive disorder mediated by autoantibodies and complement activation, has traditionally been managed with corticosteroids and splenectomy; however, a considerable proportion of patients experience relapse or refractory disease. In recent years, advances in the understanding of its pathogenesis have shifted the therapeutic paradigm from nonspecific immunosuppression to mechanism-based targeted therapy, including B-cell– and plasma cell–targeted agents, complement inhibitors, and cellular therapies (Table 4). Emerging innovative approaches, such as next-generation CAR-T and CAR-NK cell therapies targeting B-cell and plasma cell compartments, as well as gene-editing technologies, may further expand the possibility of durable immune reset and curative treatment in selected patients. Future efforts should focus on precision stratification, rational combination strategies, long-term safety evaluation, and elucidation of resistance mechanisms, as well as biomarker-guided personalized treatment approaches. Ultimately, these strategies aim to transition from empirical immunosuppressive therapy to mechanism-based precision medicine.

**Table 4:** Systematic comparison of targeted therapies for relapsed or refractory AIHA

Therapeutic strategy	Representative agents	Best-supported subgroup	Efficacy profile	Key safety considerations
Anti-CD20 B-cell depletion	Rituximab	Relapsed wAIHA; CAD/cAIHA requiring B-cell clone control; earlier use when steroid toxicity is expected	Established second-line option; in wAIHA, reported ORR about 79% and CR about 40%; in CAD, rituximab-based regimens provide more durable disease control than corticosteroids.	Infusion reactions and infection risk; delayed onset makes it less suitable as sole therapy for immediately life-threatening hemolysis. Relapse may occur because long-lived plasma cells are not eliminated.
Syk inhibition	Fostamatinib; sovleplenib	Relapsed or refractory wAIHA with Fcγ receptor-mediated macrophage phagocytosis; patients needing oral steroid-sparing therapy	Rapid hemoglobin improvement has been observed. Sovleplenib showed a 67% week-24 ORR and 48% sustained hemoglobin remission; fostamatinib showed clinical activity but a modest placebo-adjusted benefit in the phase III FORWARD trial.	Generally manageable; common concerns include diarrhea, fatigue, hypertension, and anemia events. Blood pressure and gastrointestinal intolerance require monitoring.
BTK inhibition	Ibrutinib; orelabrutinib; rilzabrutinib; zanubrutinib	Relapsed/refractory wAIHA, especially BCR-driven disease or AIHA associated with lymphoproliferative disorders	Early data suggest rapid responses in case reports and phase II studies, including 64% ORR with rilzabrutinib and preliminary responses with orelabrutinib and zanubrutinib.	Evidence remains limited and mostly early-phase. Potential risks include cytopenia, bleeding tendency, infection, and off-target toxicity, which may vary among generations of BTK inhibitors.
PI3Kδ inhibition	Parsaclisib	Previously treated, prednisone-dependent, or refractory wAIHA with autoreactive B-cell activation	Phase II data showed early hemoglobin increases, 75% ORR at 12 weeks, 50% CR, and steroid-sparing effects in prednisone-dependent patients.	Class-related risks include infection, diarrhea/colitis, hepatic enzyme elevation, and immune-mediated toxicity; longer follow-up is needed.
Complement inhibition	Pegcetacoplan; iptacopan; sutimlimab; pegcetacoplan, iptacopan, ANX005, pozelimab, vemircopan	CAD/cAIHA with classical complement activation; selected wAIHA with prominent C3 deposition or complement-amplified hemolysis	Most compelling in cAIHA/CAD, where complement is the dominant effector pathway. Sutimlimab rapidly improves hemoglobin and hemolysis markers in CAD. In wAIHA, pegcetacoplan improved hemolysis markers and transfusion independence in a small study, but benefit is likely restricted to complement-positive cases.	Vaccination and infection-prevention strategies are essential. Breakthrough hemolysis may occur if upstream antibody production persists. These agents control hemolysis but may not eradicate the pathogenic B-cell clone.
Plasma-cell targeting	Bortezomib; daratumumab	Refractory or multirefractory wAIHA/CAD, especially after rituximab failure or suspected long-lived plasma-cell-driven autoantibody production	Bortezomib-rituximab produced high ORR in small studies; daratumumab has induced responses in rituximab-refractory cases. These agents are most rational when CD20-directed therapy fails.	Infection, cytopenias, neuropathy with proteasome inhibition, hypogammaglobulinemia, and infusion reactions require careful monitoring. Evidence is based mainly on small cohorts and case reports.
BAFF/FcRn and cellular therapy	Ianalumab; nipocalimab; CD19 CAR-T cells	Highly refractory disease, steroid-dependent disease, or cases requiring deep immune reset; CAR-T currently investigational	Ongoing trials may clarify the roles of BAFF blockade and FcRn inhibition. CD19 CAR-T has produced deep drug-free remissions in relapsed wAIHA, but experience remains limited.	Need careful selection because of infection risk, prolonged B-cell depletion, hypogammaglobulinemia, CRS/ICANS for CAR-T, and uncertain long-term safety.

## Data Availability

Not applicable.

## Abbreviations

AIHA: Autoimmune hemolytic anemia
BAFF: B-cell activating factor
wAIHA: warm autoimmune hemolytic anemia
BAFF-R: B-cell activating factor receptor
BCMA: B-cell maturation antigen
BCL-2: B-cell lymphoma 2
BCR: B-cell receptor
BTK: Bruton tyrosine kinase
CAD: Cold agglutinin disease
CAR-T: Chimeric antigen receptor T-cell
CAS: Cold agglutinin syndrome
cAIHA: Cold autoimmune hemolytic anemia
CLL: Chronic lymphocytic leukemia
CR: Complete remission
CRS: Cytokine release syndrome
DAT: Direct antiglobulin test
DVd: Daratumumab: bortezomib: and dexamethasone
Fc: Fragment crystallizable
FcγRIIb: Fc gamma receptor IIb
FcRn: Neonatal Fc receptor
Hb: Hemoglobin
HLA-DRB5: Human leukocyte antigen-DRB5
HSCT: Hematopoietic stem cell transplantation
ICANS: Immune effector cell-associated neurotoxicity syndrome
IFN-γ: Interferon-gamma
Ig: Immunoglobulin
IL-6: Interleukin-6
ITP: Immune thrombocytopenia
LDH: Lactate dehydrogenase
mAIHA: Mixed autoimmune hemolytic anemia
mTOR: Mechanistic target of rapamycin
ORR: Overall response rate
PCH: Paroxysmal cold hemoglobinuria
PI3K: Phosphoinositide 3-kinase
PI3Kδ: Phosphoinositide 3-kinase delta
PNH: Paroxysmal nocturnal hemoglobinuria
PR: Partial remission
RBC: Red blood cell
Syk: Spleen tyrosine kinase
TGF-β: Transforming growth factor-beta
Th1: T helper 1 cell
Th17: T helper 17 cell
TNF-α: Tumor necrosis factor-alpha
Treg: Regulatory T cell
